# Supplementary material for: Prevalence of metabolic syndrome and associated factors among patients with chronic Chagas disease
Source: PLoS One. 2021 Apr 2;16(4):e0249116. doi: 10.1371/journal.pone.0249116 (PMC8018626; doi:10.1371/journal.pone.0249116)
Supplement: Attachment — Submitted filename: Review_ PLOS ONE_10242020_Prevalence of metabolic syndrome and associated factors among patients with chronic.docx [file pone.0249116.s001.docx]

**Prevalence of metabolic syndrome and associated factors among patients with chronic**

**Chagas disease**

**1. Overview of the manuscript:** The manuscript describes the prevalence of the metabolic syndrome and its components in patients with chronic Chagas disease and the possible increase of the MetS in individuals that migrated from rural to urban centers. The authors reported a high prevalence (about 40%) of MetS in patients with chronic ChD which is greater than the general population reported in other studies that were conducted in Brazil and worldwide. However, it is important to take in consideration the age of the population, degree of education, the study design etc. The Metabolic syndrome is normally associated with increased risk for cardiovascular disease that it is directly related to the lifestyle. The rates can be different based on the country, individual sites, study criteria classification and the characteristics of the studied population. The authors evaluated the metabolic syndrome and its components using clinical electrocardiographic, echocardiographic, and digestive exams. No pathological aspects of the Chagas diseases were mentioned, especially in the patients that developed the MetS.

Overall, this seems to be a well conducted study, the results are generally clearly presented, and the paper is well written. The purpose of this study is relevant. **There are not major issues in interpreting the study data. However, in order to improve the quality of the manuscript, the issues outlined below should be considered before publication.**

**2. Introduction section**: For a better comprehension of the study, below are a few suggestions:

a. It will be better if the manuscript had line numbers and page references.
b. In the first paragraph of the introduction, reference 1 refers to an article from 2015 and reference 2 refers to an article from 2018 (Médecins Sans Frontières). I suggest using more updated information, such as the WHO website.
c. The MetS is mentioned for the first time in the second paragraph. I suggest reviewing this paragraph and better describe the syndrome, including a short history of the background and how there has been varying definitions until the WHO provided the currently recognized international definition.

**3. Methods section**: There are few suggestions which follow:

1. If the patients are from different sites or a specific site in Brazil, it would beneficial to provide a map of the study site(s).
2. I would recommend separating the methods into:
a. Study design, period and population
b. Sample size.
c. Study procedure or Inclusion criteria.
d. Ethical considerations
e. Clinical follow up
f. Clinical form of ChD
g. Evaluation of nutritional status
h. Socioeconomic data and lifestyle
i. Data management and statistical analysis

**4. Results section**:

a. Please specify in the text where this data can be found (table?) “**The overall mean age was 60.7 years, with 56.2% women. There was a predominance of mulatto race (59.8%) and most participants had less than 9 years of schooling (67.3%)”**. If it is listed in the table 1, please review the values, they are different from table 1.

b. The percentage regarding “**The prevalence of hypertension, dyslipidemia, obesity and diabetes were 67.3% (n=243), 53.5% (n=193), 25.8% (n=93) and 21.7% (n=78), respectively (Figure 1)”.** My calculation was 21.6% instead of 21.7%.

c. I suggest specifying the % for each of the variables described in the last paragraph of page 15. It will maintain consistency with the other description of the results.

**5. Discussion section:** this section is in line with the results; the authors have discussed the data and brought the arguments. Few suggestions which follow:

a. **“The main finding of the present study was a high prevalence (about 40%) of MetS in patients with chronic ChD that was greater than in the general population in other studies conducted in Brazil and worldwide** (ref 10)”. Please add more references.

b. **“Therefore, a possible explanation to the high prevalence of MetS observed in our**

**study is the high percentage of 61% of the participants aging >60 years.”** It was previously mentioned in the beginning of this paragraph. I suggest removing it.

c. **“The educational level was also an important variable related to MetS in our study, wherein those with >12 years of education had 64% lower odds to develop MetS in comparison to those with < 9 years (%)”.** Please add the %.
d. **“Surprisingly, carbohydrate consumption was a protective factor for the development of MetS in our study, although not reaching statistical significance in the multivariate model (OR 0.99; 95%CI 0.99 to 1.00; p=0.06)”.** I did not find this p=0.06 in the table 2.
