# Supplementary material for: Prevalence of metabolic syndrome and associated factors among patients with chronic Chagas disease
Source: PLoS One. 2021 Apr 2;16(4):e0249116. doi: 10.1371/journal.pone.0249116 (PMC8018626; doi:10.1371/journal.pone.0249116)
Supplement: Attachment — Submitted filename: Response Letter PlosOne.R1.docx [file pone.0249116.s002.docx]

**Response to Editor**

**Manuscript ID: PONE-D-20-16190**

**Title: Prevalence of metabolic syndrome and associated factors among patients with chronic Chagas disease**

Thank you very much for giving us the opportunity to address the issues raised by the reviewers. We also thank the reviewers for their diligent work reviewing the manuscript. We reviewed the manuscript in order to meet reviewers’ suggestions. Changes were addressed point by point below and marked in yellow in the manuscript. We hope the paper is clearer now and suitable for publication at Plos One. Please, feel free to contact me in case of any additional doubts.

Sincerely yours,

Mauro Felippe Felix Mediano, PhD

Journal Requirements:

When submitting your revision, we need you to address these additional requirements.

1. Please ensure that your manuscript meets PLOS ONE's style requirements, including those for file naming. The PLOS ONE style templates can be found at

<https://journals.plos.org/plosone/s/file?id=wjVg/PLOSOne_formatting_sample_main_body.pdf> and

<https://journals.plos.org/plosone/s/file?id=ba62/PLOSOne_formatting_sample_title_authors_affiliations.pdf>

*Response:* *The manuscript was corrected to meet the Plos One`s style requirements.*

2. Thank you for stating in your manuscript test that "All participants received information about the goals and procedures of the study and agreed to participate by signing an informed consent form." Please also add this information to your ethics statement in the online submission form.

*Response:* *This information was included in the ethics statement in the online form.*

3. Please include additional information regarding the survey or questionnaire used in the study and ensure that you have provided sufficient details that others could replicate the analyses. For instance, if you developed a questionnaire as part of this study and it is not under a copyright more restrictive than CC-BY, please include a copy, in both the original language and English, as Supporting Information.

*Response:* *In the development of the present study, we used a physical form to store the information and the data were released on a digital platform Research Electronic Data Capture (REDCap). All the questionnaires included in the present study are validated tools widely used in the literature. The specifications on the variables included in the study are described in the methods section.*

4. Please include a caption for figure 1.

*Response:* *We included the caption in figure 1, as suggested.*

**Additional comments to address:**

Abstract: Line 9 starts with “Most participants were women (56.2%)”. The use of the term “Most” can be replaced with a different term as women participants are only slightly higher than men.

*Response:* *The sentence has been replaced to "About half were female (56.2%)".*

Data analysis: Since there were multiple comparisons, wondering if there was an attempt to correct for this by adjusting the P values and significance threshold?

*Response: We appreciate the reviewer comment. Historically, correcting the p-values for multiple tests began with post-hoc testing following ANOVA. In theory, this rationale is also applied to all statistical procedures with multiple comparisons. However, considering that the number of tests performed in multiple regression analysis are usually high, it would be very difficult to declare a statistically significant result considering corrections for multiple tests. Therefore, the use of statistical tests to correct for multiple comparisons in multiple regression models is not common. A possible strategy to deal with multiple comparisons is to change the significance level to lower values (0.01, for example). In our study,* *all variables maintained in the multivariate model had p-values <0.01. Therefore,* *our results were consistent even if setting the probability of type 1 error to 1%. The multiple comparison issue was included as a possible limitation in the last paragraph of the discussion.*

Results and Discussion: Majority of the study participants were from Mulatto ethnic group. It will be useful to discuss whether a high prevalence (40%) of MetS in patients with chronic ChD identified in this population is generalizable to Brazilian population broadly.

*Response:* *The high prevalence (40%) of MetS found in the study can only be applied to the ChD population since they have specific characteristics. We included this information in the limitation paragraph of the discussion section.*

In the last part of the discussion it will be useful to state how this study finding can be helpful in developing preventive strategies to minimize the risk of MetS in ChD patients.

*Response:* *Thanks for the suggestion. We included some strategies that can be used to prevent and minimize the risk of MetS in patients with ChD.*

Reviewers' comments:

**Introduction section**:

a. It will be better if the manuscript had line numbers and page references.

*Response: The page and line numbers were included in the manuscript.*

b. In the first paragraph of the introduction, reference 1 refers to an article from 2015 and reference 2 refers to an article from 2018 (Médecins Sans Frontières). I suggest using more updated information, such as the WHO website.

*Response: The reference was updated, as suggested by reviewer.*

c. The MetS is mentioned for the first time in the second paragraph. I suggest reviewing this paragraph and better describe the syndrome, including a short history of the background and how there has been varying definitions until the WHO provided the currently recognized international definition.

*Response: We have included a paragraph better describing the MetS, including the current WHO definition.*

**Methods section**

1. If the patients are from different sites or a specific site in Brazil, it would beneficial to provide a map of the study site(s).

*Response:* *The patients included in the study have different origins, most of whom are from rural areas in Brazil. However, at the time the study was conducted, all participants lived in the metropolitan region of the state of Rio de Janeiro. We included this specification in the study design, period and population section.*

2. I would recommend separating the methods into:
a. Study design, period and population
b. Sample size.
c. Study procedure or Inclusion criteria.
d. Ethical considerations
e. Clinical follow up
f. Clinical form of ChD
g. Evaluation of nutritional status
h. Socioeconomic data and lifestyle
i. Data management and statistical analysis

*Response: The methods were separated into new subsections.*

**Results section**

a. Please specify in the text where this data can be found (table?) “The overall mean age was 60.7 years, with 56.2% women. There was a predominance of mulatto race (59.8%) and most participants had less than 9 years of schooling (67.3%)”. If it is listed in the table 1, please review the values, they are different from table 1.

*Response: These data represents the major characteristics of the overall study sample and were included only in the manuscript text to avoid repeated data presentation.*

b. The percentage regarding “The prevalence of hypertension, dyslipidemia, obesity and diabetes were 67.3% (n=243), 53.5% (n=193), 25.8% (n=93) and 21.7% (n=78), respectively (Figure 1)”. My calculation was 21.6% instead of 21.7%.

*Response: We apologize for this mistake. The correct value is 21.6%. This information was corrected in the manuscript and Figure 1.*

c. I suggest specifying the % for each of the variables described in the last paragraph of page 15. It will maintain consistency with the other description of the results.

*Response:* *In order to meet the reviewer`s request, we include the values for each variable described in the suggested paragraph.*

**Discussion section:**

a. “The main finding of the present study was a high prevalence (about 40%) of MetS in patients with chronic ChD that was greater than in the general population in other studies conducted in Brazil and worldwide (ref 10)”. Please add more references.

*Response: We have included three additional references in this paragraph.*

b. “Therefore, a possible explanation to the high prevalence of MetS observed in our study is the high percentage of 61% of the participants aging >60 years.” It was previously mentioned in the beginning of this paragraph. I suggest removing it.

*Response:* *We removed this information from the text.*

c. “The educational level was also an important variable related to MetS in our study, wherein those with >12 years of education had 64% lower odds to develop MetS in comparison to those with < 9 years (%)”. Please add the %.

*Response:* *The percentage included in this sentence refers to the protective effect of >12 years of education (64% lower odds of having MetS). We rephrased the sentence to improve clarity.*

d. “Surprisingly, carbohydrate consumption was a protective factor for the development of MetS in our study, although not reaching statistical significance in the multivariate model (OR 0.99; 95%CI 0.99 to 1.00; p=0.06)”. I did not find this p=0.06 in the table 2.

*Response: The p-value of 0.06 refers to the multivariate analysis; therefore, it is not shown in Table 2 that only includes values for the univariate analysis. The p-value of 0.06 (for carbohydrate) was also not seen in the Table 3 because it was not statistically significant.*
